# Consensus on the Management of the Clinical Challenges of Venous Thromboembolism in Special Situations

**DOI:** 10.1055/a-2595-1811

**Published:** 2025-05-13

**Authors:** Cristina Marzo, Teresa Solanich, Dolors Tassies, Elena Pina, Maite Antonio Rebollo, Enrique Gallardo, Sonia Serradell, Marta Merino, Marina Carrasco, Albert Tugues

**Affiliations:** 1 Hematology and Hemotherapy Department, Hospital Universitari Arnau de Vilanova, Lleida, Catalunya, Spain; 2 Vascular Surgery Department, Hospital Universitari Parc Taulí, Sabadell, Spain; 3 Hemotherapy and Hemostasis Department, Hospital Clinic Barcelona, Barcelona, Catalunya, Spain; 4 Thrombosis and Haemostasis Department, Hospital Universitari Bellvitge, Hospitalet de Llobregat, Barcelona, Spain; 5 Oncogeriatrics Unit, Institut Català d'Oncologia, Barcelona, Spain; 6 Oncology, Parc Taulí Hospital Universitari, Sabadell, Barcelona, Spain; 7 Institut d'Investigació i Innovació Parc Taulí I3PT, Universitat Autònoma de Barcelona, Sabadell, Barcelona, Spain; 8 Medical Oncology Department, Vall d'Hebron University Hospital, Barcelona, Spain; 9 Medical Affairs Thrombosis, LEO Pharma Spain, Barcelona, Spain; 10 Unitat d'Hemostasia i Trombosi, Servei d'Hematologia, Hospital de la Santa Creu i Sant Pau, Barcelona, Spain; 11 Hematology and Hemotherapy Department, Hospital Universitario Arnau de Vilanova de Lleida, Barcelona, Spain

**Keywords:** heparins, malignancy, obesity, pregnancy, venous thrombosis

## Abstract

Venous thromboembolism (VTE) can present different challenging situations for which high-quality evidence to guide optimal preventive and therapeutic management is lacking and for which clinical practice guidelines have not established solid recommendations. The aim of this article is to achieve consensus on a proposal of action for the clinical management of complex, clinically relevant situations with a low level of evidence which generate great uncertainty—the duration of VTE treatment and the role of thrombus recanalization, the prevention of VTE within the context of pregnancy, management of anticoagulant treatment in patients with VTE and special characteristics, such as renal insufficiency and obesity, the therapeutic management of pluripathological and polymedicated older patients with VTE, and finally, primary ambulatory thromboembolic prevention in cancer patients. This consensus article arose from a collaboration of experts in VTE from different medical specialties.

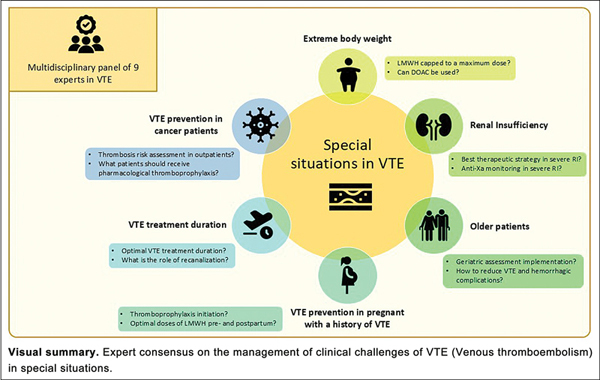

## Introduction

Venous thromboembolism (VTE) is an increasingly more prevalent disease and is a common cause of morbidity and mortality. It is the third most frequent cause of cardiovascular death, following coronary disease and ischemic stroke, leading to more than 500,000 deaths in the European Union and up to 300,000 deaths in the United States each year. 1

The etiology of VTE is multifactorial. Some patients may have a hereditary predisposition to thrombosis and/or VTE may be triggered by transitory and/or persistent risk factors. In approximately half of the cases, VTE occurs in the absence of known risk factors.

Anticoagulant therapy is the pillar of VTE treatment. The situations presented in daily clinical practice are of increasingly greater complexity due to the pluripathology of the patients and because anticoagulant treatment is currently inevitably associated with greater risk of bleeding. The incidence of bleeding in patients receiving anticoagulation treatment is approximately 10 to 20%, depending on the type of anticoagulant and the specific comorbidities of the patients. 2

The management of the prevention and treatment of VTE can present some challenging situations for which high-quality evidence is lacking for guiding optimal management in daily clinical practice. This clinical ambiguity between the risk of thrombosis and the risk of bleeding is challenging for clinicians.

The article aims to address questions related to the management of anticoagulant therapy in the treatment and prevention of VTE for which scientific evidence is limited and establish some recommendations of action to facilitate clinical decision-making.

## Methodology

With the aim of obtaining consensus for a proposal of action on the clinical management of complex situations with low level of evidence, a multidisciplinary panel of nine experts in VTE was created including the specialties of Hematology, Internal Medicine, Medical Oncology, and Vascular Surgery.

Under the coordination of one expert hematologist, in June 2023, this panel of experts met to discuss and agree on which scenarios of VTE needed to be developed, taking into account the clinical interest and relevance in patients with VTE. The following six situations of controversial clinical management were selected: (1) the duration of VTE treatment and the role of thrombus recanalization; (2) the prevention of VTE within the context of pregnancy, the management of anticoagulant treatment in patients with VTE and special characteristics such as (3) renal insufficiency and (4) obesity; (5) the therapeutic management of older patients with pluripathology and polypharmacy with VTE; and finally (6) primary ambulatory thromboembolic prevention in cancer patients. From each of the situations, 2-3 specific questions were formulated, and the distribution of the situations along with their respective questions was agreed upon among the panelists. A comprehensive electronic literature search using the PubMed and Google Scholar databases was performed to identify all relevant studies and guidelines from 2000 to June 2023. Only human studies published in English language were included. The search terms and the medical subject headings (MeSH) used were: “venous thromboembolism” AND “treatment duration,” “venous thromboembolism” AND “recanalization” AND (“residual vein thrombosis” OR “residual vein obstruction”), (“prophylaxis” OR “thromboprophylaxis”) AND “pregnancy” AND “venous thromboembolism,” “venous thromboembolism” AND (“renal insufficiency” OR “renal impairment”), “anti-Xa monitoring” AND (“heparin” OR “low molecular weight heparin”), “ venous thromboembolism” AND (“extreme body weight” OR “obesity”), “venous thromboembolism” AND (“elderly” OR “aged” OR “frail elderly”), (“thromboprophylaxis” OR “prophylaxis”) AND “ambulatory” AND “cancer” AND “placebo” AND (“thrombosis” OR “survival”). The authors who conducted the search took advantage of the references of these articles to manually search for other relevant publications.

After carrying out the project, the experts met again to share the recommendations taking into account the severity of the clinical problem, the magnitude of risks and benefits, and the quality of evidence and feasibility. The level of evidence and the grade of recommendation were determined using an adaptation of the classification system of the Infectious Diseases Society of America 3 ( Table 1 ). All the experts voted for or against each of the recommendations and after calculating the results, if there was <75% consent or >20% dissent regarding an item in particular, the subject was discussed and recirculated among the panelists until a unanimous consensus was reached.

**Table 1:** Levels of evidence and grades of recommendation (adapted from the Infectious Diseases Society of America – United States Public Health Service Grading System)

**Levels of evidence**	
I	Evidence from at least one large randomized, controlled trial of good methodological quality (low potential for bias) or meta-analyses of well-conducted randomized trials without heterogeneity.
II	Small randomized trials or large randomized trials with suspicion of bias (lower methodological quality) or meta-analyses of such trials or of trials with demonstrated heterogeneity.
III	Prospective cohort studies.
IV	Retrospective cohort studies or case-control studies.
V	Studies without control group, case reports, expert opinions.
**Grades of recommendation**	
A	Strong evidence of efficacy with substantial clinical benefits, strongly recommended.
B	Strong or moderate evidence of efficacy but with limited clinical benefits, generally recommended.
C	Insufficient evidence of efficacy or benefits does not outweigh the risk or the disadvantages (adverse events, costs, etc.), optional.
D	Moderate evidence against efficacy or for adverse outcomes, generally not recommended.
E	Strong evidence against efficacy or for adverse outcomes, never recommended.

## Results

### Duration of VTE Treatment and the Role of Thrombus Recanalization

#### What is the Recommended Duration of VTE Treatment?

The following factors must be considered to decide on the duration of anticoagulant treatment in a patient presenting a VTE event: the etiology of the thrombosis, the localization of the thrombus, the number of episodes, the risk of recurrence, the risk of bleeding, and the preferences of the patient in relation to treatments of equal efficacy and safety.

Clinical guidelines distinguish the duration of anticoagulant treatment based on whether the thrombosis is provoked or unprovoked. In the group of provoked VTE, the duration of treatment depends on whether the transitory risk factor is strong, moderate, or weak, or whether the risk factor is persistent. 4 Table 2 shows the different risk factors. In the group of unprovoked VTE, the risk of recurrence is greater and determines the duration of treatment to avoid recurrence. In the latter group, the risk of recurrence is 15% in the first year and 40.5% at 5 years. In the provoked VTE group, the risk of recurrence at 1 year is 6.6%, and 16.1% at 5 years. Tables 3 and 4 show the different anticoagulant therapies available and the recommended duration of treatment based on the factors analyzed. 5 6

**Table 2:** Risk factors of venous thromboembolism

Strong risk factors (odds ratio >10)	Moderate risk factors (odds ratio 2–9)	Weak risk factors (odds ratio <2)
• Major surgery (orthopedic and neurological)/major trauma • Recent (<3 months) hospitalization for acute heart disease • Previous venous thromboembolism • Antiphospholipid syndrome • Active cancer (depends on type and stage/chemotherapy)	• Arthroscopic knee surgery • Venous catheters • Oral contraception/hormone replacement therapy • Pregnancy or postpartum period • Inflammatory and autoimmune diseases • Infections • Congestive heart or respiratory failure • Genetic thrombophilia • Superficial vein thrombosis (>3 cm from SFJ or PJ and >5 cm length) • Stroke with residual hemiparesis/hemiplegia	• Bed rest (>3 days)/immobility (prolonged sitting position, i.e., travel) • Age • Obesity • Superficial vein thrombosis • Varicose veins/chronic vein insufficiency • Laparoscopic surgery

Abbreviations: PJ, popliteal junction; SFJ, sapheno-femoral junction.

Source: Table adapted from Mazzolai et al. 6

**Table 3:** Anticoagulant treatment options: parenteral, direct oral anticoagulants, and vitamin K antagonists

**A. Parenteral (LMWH and heparinoids)**		
	**Posology**	
**Enoxaparin**	1 mg/kg/12 h or 1.5 mg/kg/day	
**Tinzaparin**	175 IU/kg/day	
**Bemiparin**	115 IU/kg/day	
**Dalteparin**	100 IU/kg/12 h or 200 IU/kg/day	
**Fondaparinux**	<50 kg: 5 mg/24 h, 50–100 kg: 7.5 mg/24h, >100 kg: 10 mg/24 h	
**B. Direct oral anticoagulants (DOAC)**		
	**Posology**	
	**Acute phase**	**Long term**
**Rivaroxaban**	15 mg/12 h × 21 days	20 mg/24 h (10 mg/24 h) a
**Apixaban**	10 mg/12 h × 7 days	5 mg/12 h (2.5 mg/12 h) a
** Edoxaban bc**	60 mg/24 h (5 days of previous LMWH)	60 mg/24 h
** Dabigatran c**	150 mg/12 h (5 days of previous LMWH)	150 mg/12 h
**C. Vitamin K antagonists**		
	**Posology**	
	**Acute phase**	**Long term**
**Acenocumarol** **Warfarin**	No isolated use in initial phase. Overlap with LMWH until INR in range	Individualized dose for INR between 2 and 3

Abbreviations: INR, international normalized ratio; LMWH, low molecular weight heparin.

^a^ Dose for extended treatment.

b Dose of 30 mg/day with one or more of the following clinical factors: creatinine clearance 15–50 mL/min, low body weight ≤60 kg, or concomitant use of any glycoprotein P (P-gp) inhibitors.

c Requires previous parenteral anticoagulant treatment of at least 5 days.

**Table 4:** Duration of anticoagulant treatment

**3 months**	• Provoked VTE due to minor risk factor • Distal DVT
**6 months**	• Unprovoked VTE • Provoked VTE due to major risk factor • Proximal DVT
**12 months**	• Evaluate according to D-dimer values, risk of recurrence, and risk of bleeding
**Indefinite**	• Recurrences • Persistence of major risk factors (i.e., active cancer)

Abbreviations: DVT, deep vein thrombosis; VTE, venous thromboembolism.

Among the different therapeutic options, of note are the studies that analyzed the safety of subcutaneous injection of low molecular weight heparin (LMWH) in acute and long-term treatment compared with oral anticoagulation, with both being equally effective and safe. 7

In non-oncological patients, the guidelines recommend treatment with direct oral anticoagulants (DOACs) as the first choice and treatment with LMWH as the option of initiation of parenteral treatment. In cancer patients LMWH and DOAC are indicated for the initial phase and long-term treatment (up to 6 months). Nonetheless, DOACs are associated with an increased risk of bleeding compared with LMWH, especially in non-resected gastrointestinal (GI) or genitourinary (GU) tumors; therefore, the international guidelines recommend LMWH over DOAC in increased bleeding risk situations such as GI/GU tumors with a high bleeding risk, severe renal insufficiency, unstable clinical situations (low platelet count, nausea, and vomiting), and drug interactions. 6 8

In non-oncological patients, treatment with LMWH can be continued or switched to oral anticoagulation. In the study by Hull et al, the results were analyzed based on the continuation of treatment with tinzaparin or a switch to warfarin, and it was concluded that prolonged treatment with LMWH improves thrombus recanalization, and patients present fewer symptoms of post-thrombotic syndrome. 9

The guidelines of the European Society for Vascular Surgery state that, according to a Cochrane review, LMWH and vitamin K antagonists (VKAs) are equally effective and safe in the treatment of provoked and unprovoked venous thrombosis. The review considered that 3-month treatment with LMWH is a good alternative to a switch to VKA in provoked venous thrombosis due to the dose adjustments required in VKA treatment. In these cases, patient preferences as well as healthcare costs are very important. 4

The Home-Life study compared 3-month treatment with tinzaparin versus tinzaparin for 5 days followed by warfarin. Patient satisfaction with the treatment and the presence of symptoms and signs of post-thrombotic syndrome (PTS) were analyzed, concluding that the grade of satisfaction was greater in the LMWH group ( *p* = 0.0024). A lower incidence of post-thrombotic syndrome and ulcers was also found in the group receiving LMWH. There were no differences between the groups in regard to recurrence, mortality, or bleeding. 10

##### Recommendations

- In provoked VTE with a major transient risk factor, 3 months of anticoagulation treatment is recommended (Grade I, A recommendation).
- In patients who cannot receive a DOAC, a 3-month treatment with LMWH could be considered over VKA, as LMWH was found to reduce the PTS incidence and to show a higher patient satisfaction compared with VKA (Grade II, C recommendation).
- In unprovoked VTE with low and moderate bleeding risk, extended anticoagulation beyond 3 months, with periodic reassessment of bleeding risk, is recommended (Grade I, A recommendation).
- In patients with VTE within the context of malignant neoplasia, anticoagulant treatment of at least 6 months is recommended (Grade I, A recommendation).
- LMWH is preferred over DOAC for initial and long-term treatment in cancer patients with GI or GU tumors, high risk for gastrointestinal bleeding, unstable clinical situations, such as low platelet count, nausea, and vomiting, severe renal insufficiency, and a risk of expected drug interactions with the anti-cancer therapy (Grade I, B recommendation).

#### What Role Does Thrombus Recanalization Play in Deciding the Optimal Duration of Anticoagulant Treatment? Should a Duplex Ultrasound Assessment be Performed?

First, it is necessary to define residual venous thrombosis (RVT). In the literature there are different ultrasound (US) criteria. A review described the most common US criteria. The Prandoni criterion is a diameter in the transverse plane of greater than 2 mm in a US study or greater than 3 mm in three consecutive US studies. Siragusa considers the presence of RVT when a thrombus occupies more than 40% of the vessel lumen, and a third criterion is the presence of residual thrombus with a thickness of more than 1 mm. 11

The same review included a study demonstrating that the presence of RVT is a risk factor of VTE recurrence in patients with cancer. 11

The Prolong Study analyzed the presence of isolated RVT or associated with D-dimer as a risk factor of recurrence after stopping anticoagulant treatment in unprovoked thrombosis and concluded that the presence of elevated D-dimers at 1 month after treatment finalization is a risk factor of recurrence, but not the presence of RVT. 12

Another study analyzed whether RVT is a risk factor of recurrence in unprovoked thrombosis after treatment with anticoagulation for 3 months. It concluded that RVT is a strong predictive factor if detected at 3 months after the diagnosis of thrombosis (hazard ratio [HR] 2.7; confidence interval [CI] 1.11–4.25), but it is not a significant risk factor if detected at between 3 and 6 months or more than 6 months after diagnosis (HR 1.14 and 1.19, respectively). 13

A study published in 2023 analyzed not only the role of RVT as a risk factor of recurrence but also as a risk factor of PTS, arterial events, and cancer. It found that RVT was associated with the presence of PTS (HR 1.66, CI 1.19–2.32) but not with recurrence or cancer. 14

In another systematic review, it was reported that the presence of RVT is not a predictor of recurrence of VTE in patients with unprovoked VTE, similar to what was found in the Reverse Cohort Study and in the EXTENDED Cancer-DACUS study. 11 15 16

##### Recommendations

- The presence of RVT is not a strong risk factor of recurrence and should, therefore, not condition the duration of treatment (Grade I, B recommendation).
- The indication for performing a duplex ultrasound on finalizing anticoagulant treatment is not aimed at prolonging treatment if RVT is detected, but rather, in the case of symptomatic recurrence, it is performed to determine if this is a sequela or a new thrombotic episode (Grade I, B recommendation).

### Prophylaxis of Venous Thromboembolism (VTE) in Pregnancy

#### When Should Thromboprophylaxis with Low Molecular Weight Heparin (LMWH) be Initiated in Pregnant Women with a History of VTE?

A history of previous VTE, especially unprovoked or related to exposure to estrogens, is the most important individual risk factor of recurrence in pregnancy. 17 This risk is estimated to be 10.9% compared with 3.7% in non-pregnant women (relative risk [RR] 3.5; 95% CI 1.6–7.8) if prophylaxis is not given. 18 The time at which prophylaxis should be initiated depends on different factors.

In pregnant women with a history of VTE who are not receiving long-term anticoagulation, the guidelines recommend that all of these women should receive postpartum thromboprophylaxis with LMWH. 19 20 21 22 In the guidelines of the American Society of Hematology (ASH), the grade of recommendation is strong, with low certainty of evidence regarding its effects. 19 Thromboprophylaxis should continue up to at least 6 weeks postpartum (level of evidence 2B and Grade B recommendation). 20 21

With respect to thromboprophylaxis before delivery, the guidelines recommend that it be performed throughout the antepartum period if the VTE was idiopathic, associated with a hormonal risk factor (pregnancy, combined hormonal contraceptives) or thrombophilia, or was recurrent. 19 20 21 22 23 The grades of recommendation are C, 21 strong recommendation with low certainty of evidence of the effects, 19 and grade 2C. 20 On the other hand, the guidelines of the American College of Obstetricians and Gynecologists (ACOG) 23 and those of the German Society of Thrombosis and Hemostasis 22 do not mention grades of recommendation.

In patients with a history of VTE associated with a major, transitory, non-hormonal risk factor and without thrombophilia or other additional risk factors, the ASH guidelines recommend against antepartum prophylaxis, 19 being a conditional recommendation with low certainty of evidence about its effects.

Likewise, when the risk of recurrence of VTE is low (isolated episode, associated with a transitory risk factor, not related to pregnancy or the use of estrogens), the guidelines of the American College of Chest Physicians (ACCP) recommend clinical monitoring instead of antithrombotic prophylaxis in the antepartum period, with a Grade 2C recommendation. 20

In general, if antepartum prophylaxis is indicated, this should be initiated as soon as possible during the first trimester and should be continued up to at least 6 weeks postpartum.

##### Recommendations

In pregnant women with a history of VTE not receiving long-term anticoagulation:

- If the VTE was associated with a major transitory risk factor and in the absence of other risk factors, prophylaxis is recommended only in the postpartum period (Grade III, B recommendation).
- If the VTE was unprovoked, related to estrogens, or recurrent, it is recommended to initiate prophylaxis with LMWH in the first trimester and maintain this until at least 6 weeks postpartum (Grade II, B recommendation).

#### What are the Optimal Doses of LMWH Pre- and Postpartum in Pregnant Women with a History of VTE?

The most adequate dose of LMWH for prophylaxis in pregnant women with a previous history of VTE who are not receiving long-term anticoagulant treatment is controversial.

The ACCP guidelines do not provide specific recommendations regarding the dose of LMWH for antepartum and postpartum prophylaxis and in both periods the prophylactic or intermediate doses of LMWH are considered without distinction. 20 For antenatal and postnatal prophylaxis, the ACOG indistinctly recommends prophylactic doses, intermediate doses, or doses adjusted to weight in patients with a history of idiopathic or estrogen-associated VTE. 19

In patients with high-risk thrombophilia, such as antithrombin deficiency, antiphospholipid syndrome, or with recurrent thrombosis (who are often receiving long-term anticoagulation), prophylaxis with LMWH at higher than standard prophylactic doses (50, 75, or 100% of the therapeutic doses) is recommended, with a Grade D 21 and III-B 24 recommendation, while hereditary thrombophilia of lower risk can be managed with standard LMWH prophylactic doses (Good Clinical Practice recommendation 21 and Grade I-A 24 ).

Some guidelines also suggest the use of intermediate doses of LMWH in obese patients or in those meeting the criteria for thromboprophylaxis and with several added risk factors. 19 20 21

The multicenter randomized clinical trial Highlow compared two doses of LMWH as prophylaxis during pregnancy in women with a history of VTE. There were no differences between the standard prophylactic dose and the prophylactic dose adjusted to weight (intermediate) of LMWH in relation to the combined incidence of antepartum and postpartum thrombotic events or in bleeding, and thus, it was concluded that the standard prophylactic doses of LMWH are adequate for prophylaxis. Nevertheless, in a post hoc analysis a lower incidence of pulmonary embolism (PE) and superficial thrombophlebitis was observed in the group of patients who received intermediate doses, at the expense of a reduction of these episodes in the postpartum period. This finding suggests that intermediate doses of LMWH may be more effective than a fixed prophylactic dose in the postpartum period, although further studies are necessary to confirm this. 25

##### Recommendations

In pregnant women with a history of VTE not receiving long-term anticoagulation therapy:

- The most adequate dose of LMWH to achieve antithrombotic prophylaxis is not well defined. Although the standard prophylactic dose of LMWH seems to be effective in antithrombotic prophylaxis ante- and postpartum (Grade II, B recommendation), it remains to be determined whether the intermediate doses might be more effective in the postpartum period.

### Treatment of VTE in Patients with Renal Insufficiency (RI)

#### What is the Best Therapeutic Strategy in Patients with Severe Renal Insufficiency (Glomerular Filtration Rate [GFR] <30 mL/min)? And in Patients with a GFR of 15–20 mL/min?

Renal insufficiency (RI) is becoming a more prevalent disease in modern society, increasing the risk of bleeding as well as thrombosis in these patients, and being a challenge in selecting the most adequate anticoagulant treatment. 26 27 Many anticoagulants, including LMWH, fondaparinux, and DOAC, are eliminated, at least in part, via the kidneys and the risk of drug accumulation and posterior bleeding increases in patients with RI. 28

Decision-making regarding the anticoagulant strategy of choice in patients with severe RI (GFR <30 mL/min) is not easy. The results of large pivotal studies support the efficacy and safety of the use of warfarin and DOAC in mild or moderate chronic kidney disease. However, there are few studies of quality on advanced chronic kidney disease and they report contradictory results. 29

This lack of scientific evidence means that the main clinical practice guidelines, which advocate for the use of DOAC over VKA in VTE (Grade 1A), include patients with a GFR <30 ml/min as an exception to this rule (Grade 5E), as is the case with the 2019 European Society of Cardiology (ESC) guidelines, which do not recommend the use of DOAC in these patients. 30 31

In the Summary of Product Characteristics (SmPC), dabigatran is contraindicated in cases with a GFR <30 mL/min and, “in any case, doses adjusted for the GFR should be evaluated in the DOACs which allow their use in patients with a GFR <30 mL/min” with “and when a DOAC is chosen whose product label allows its use with GFRs below 30 ml/min, an adjusted dose based on GFR should be considered” (apixaban, rivaroxaban, and edoxaban). 32 33 34 35

In the case of the LMWH, most SmPC recommend adjusting the dose with a GFR <30 mL/min, except for tinzaparin, which presents less dependence on renal excretion and does not accumulate until a GFR of 20 mL/min. 36 37 38 39 40 41 42

In cases with a GFR <15 mL/min the evidence is even less clear. DOACs are not recommended by the European Medicines Agency (EMA) in this type of patients due to the lack of scientific evidence. 32 33 34 35 One study involving a very few cases described a slight increase in the concentration of apixaban and that it was not very affected by dialysis. With these data the Food and Drug Administration recommends the use of a dose of 5 mg twice daily for nonvalvular atrial fibrillation and 2.5 mg twice daily for the prevention of thrombotic recurrence in VTE. 43

The use of LMWH with GFR <15 mL/min lacks scientific evidence but, except from nadroparin, they are not contraindicated according to the SMPCs (only “not recommended” for enoxaparin) nor do international guidelines recommend against their use in these situations. 31 38 39 40 41 42

The dose schedules recommended for the treatment of VTE by the SmPC of LMWH and DOAC based on creatinine clearance are shown in Tables 5 and 6 .

**Table 5:** Dose of LMWH for VTE treatment in renal insufficiency according to the SmPC

	Tinzaparin	Enoxaparin	Bemiparin	Dalteparin	Nadroparin
**Usual dose**	175 UI/kg/day	100 UI/kg/12 h 150 UI/kg/day	115 UI/kg/day	100 UI/kg/12 h 200 UI/kg/day	86 UI/kg/12 h 171 UI/kg/day
**CrCl 51–80 mL/min**	No dose reduction.	No dose reduction. Careful clinical follow-up is recommended.	No dose reduction. Careful clinical follow-up is recommended.	No dose reduction.	No dose reduction.
**CrCl 30–50 mL/min**	No dose reduction.	No dose reduction. Careful clinical follow-up is recommended.	No dose reduction. Careful clinical follow-up is recommended.	Adjust according to anti-Xa with significant RI (creatinine > 3 ULN).	25–33% dose reduction.
**CrCl 15–29 mL/min**	Absence of accumulation with CrCl >20 mL/min. Anti-Xa activity monitoring.	50% dose reduction. Anti-Xa activity monitoring.	25% dose reduction. Anti-Xa activity monitoring.	Adjust according to anti-Xa with significant RI (creatinine > 3 ULN).	Contraindicated.
**CrCl < 15 mL/min**	Absence of accumulation with CrCl >20 mL/min. Anti-Xa activity monitoring.	Contraindicated.	25% dose reduction. Anti-Xa activity monitoring.	Adjust according to anti-Xa with significant RI (creatinine > 3 ULN).	Contraindicated.

Abbreviations: CrCl, creatinine clearance; LMWH, low molecular weight heparin; RI, renal insufficiency; SmPC, Summary of Product Characteristics; ULN, upper limit of normal; VTE, venous thromboembolism.

**Table 6:** Dose of DOAC for VTE treatment in renal insufficiency according to the SmPC

	Apixaban	Rivaroxaban	Edoxaban	Dabigatran
**Usual dose**	10 mg/12 h (1 week) 5 mg/12 h	15 mg/12 h (3 weeks) 20 mg/day	60 mg/day	150 mg/12 h
**CrCl 51–80 mL/min**	No dose adjustment	No dose adjustment	No dose adjustment	No dose adjustment
**CrCl 30–50 mL/min**	No dose adjustment	Dose reduction: 15 mg/day If the risk of bleeding surpasses the risk of DVT and PE	Dose reduction: 30 mg/day	Dose reduction: 110 mg/12 h
**CrCl 15–29 mL/min**	No dose adjustment Use with caution	Dose reduction: 15 mg/day If the risk of bleeding surpasses the risk of DVT and PE	Dose reduction: 30 mg/day	Contraindicated
**CrCl < 15 ml/min**	Not recommended	Not recommended	Not recommended	Contraindicated

Abbreviations: CrCl, creatinine clearance; DOAC, direct oral anticoagulant; DVT, deep vein thrombosis; PE, pulmonary embolism; SmPC, Summary of Product Characteristics.

With a GFR <15 mL/min, VKAs should be considered due to their lengthy experience of use and the possibility of monitoring their activity with the INR. Nevertheless, it is difficult to maintain these patients within the therapeutic range. The SmPC of acenocoumarin contraindicates its use in patients with severe RI whenever the risk of bleeding surpasses the risk of thrombosis. 44

##### Recommendations

- In patients with RI (GFR 20–30 mL/min) both DOAC (apixaban, rivaroxaban, edoxaban) as well as LMWH adjusted according to the SmPCs or VKA can be considered. Treatment should be individualized according to the characteristics and preferences of the patients (Grade V, C recommendation).
- In patients with severe RI (GFR <15 mL/min) none of the DOACs are recommended or are contraindicated (Grade V, E recommendation), and in cases requiring anticoagulation, LMWH or VKA adjusted according to the SmPC and with close monitoring should be considered (Grade V, B recommendation).

#### Is Anti-Xa Monitoring Necessary in Patients with Severe RI to Determine the Dose Adjustment of LMWH?

There is a diversity of opinions as to whether anti-Xa monitoring is necessary in patients with severe RI treated with LMWH.

The ASH guidelines do not recommend the use of anti-Xa in patients with severe RI and instead recommend adjusting the dose of the LMWH according to the SmPC or switching to an alternative anticoagulant with less renal clearance, such as unfractionated heparin or a different LMWH (conditional recommendation based on very low certainty in the evidence about effects). 30

No statistically significant association has been found between anti-Xa levels and the antithrombotic and hemorrhagic effect. 45

Other guidelines, such as those of the ESC, 31 and the national consensus of the Spanish Society of Internal Medicine (SEMI), Spanish Society of Medical Oncology (SEOM), and Spanish Society of Thrombosis and Haemostasis (SETH), recommend evaluating the use of anti-Xa in patients with a GFR <30 mL/min. 46

Thus, the use of anti-Xa is controversial, but may be taken into account in complex patients and in long-term treatments with LMWH, although this is questionable by the guidelines.

##### Recommendations

- The use of anti-Xa in patients with severe RI is controversial and should not be routinely used (Grade V, C recommendation).

### Treatment of VTE in Patients with Extreme Body Weight

#### Should the Therapeutic Doses of LMWH be Limited to a Maximum Dose in Patients with Extreme Body Weight?

LMWHs are hydrophilic molecules with high bioavailability and intravascular distribution. There is no consensus about how to adjust the dose to weight in patients with weights greater than 100 kg. One possibility is to adjust to the real weight with the uncertainty of overdosing, and the second possibility is to limit the dose to a maximum dose, with the possible risk of underdosing. 47

The data from a prospective study suggest that treatment with tinzaparin adjusted to the real weight is safe and no accumulation of anti-Xa activity was observed in hospitalized medical patients with morbid obesity. 47 In a retrospective study of obese patients with acute VTE treated with enoxaparin, the results support a dosage strategy based on patient weight without a dose limit to guarantee the therapeutic levels of the drug. 48

The 2018 ASH guidelines on the management of the treatment of acute venous thrombosis recommend dosing LMWH according to the real weight of obese patients and not limiting the dose to a maximum weight. These recommendations have a very low level of evidence. This circumstance is not mentioned in the 2020 ASH guidelines, and thus, no recommendation is made. 30 49

The 2023 guidelines of the European Society for Medical Oncology (ESMO) suggest that in patients with extreme body weight (>120 kg or body mass index [BMI] >40 kg/m ^2^ ) the dose of LMWH should be calculated based on the real body weight of the person without establishing a maximum limit based on limited data from observational and retrospective studies. 50

With respect to the need to monitor anti-Xa in these patients, there is no consensus among the guidelines, and, at present, there is no evidence of the clinical benefits of adjusting the dose of LMWH according to anti-Xa levels. 30 51

##### Recommendations

- Taking all the above into account, the use of a dose of LMWH adjusted to the weight of the patient without establishing a maximum limit is recommended for the treatment of VTE in obese patients with weights greater than 100 kg (Grade III, B recommendation).

#### Can DOAC be Used in Patients with Extreme Body Weight?

At present, DOACs are recommended as first-line treatment for the treatment and prevention of VTE in many guidelines. 31 52

There is a lack of clinical evidence on the efficacy and safety of DOAC in the population with extreme obesity since phase 3 trials comparing different DOACs with warfarin for the treatment of VTE included relatively few patients with obesity and extreme obesity. The pharmacokinetic and pharmacodynamic data were not consistent, showing variations in this context of obesity.

The 2021 guidelines of the International Society on Thrombosis and Haemostasis (ISTH) conclude that the use of DOACs is appropriate in patients with a BMI less than 40 kg/m ^2^ or weight less than 120 kg. In cases in which the use of DOAC is unavoidable, these same guidelines suggest that obtaining peak or trough levels of these drugs may be considered reassuring, although there are currently insufficient data for clinical decision-making based on specific levels of each drug. 53

For patients with a BMI >40 kg/m ^2^ or weights >120 kg, the same guidelines consider the standard dose of rivaroxaban or apixaban, independently of the high BMI and the weight. They do not recommend the use of dabigatran or edoxaban due to the unconvincing data on dabigatran and the lack of clinical or pharmacokinetic/pharmacodynamic data on edoxaban. 53

Bariatric surgery is another consideration given the possibility of less absorption. In these patients, parenteral anticoagulation (unfractionated heparin, LMWH, or fondaparinux) in the early post-surgery phase is recommended and switching to VKA or DOAC at least 4 weeks after parenteral treatment with trough DOAC levels to verify the absorption and bioavailability of the drug. 53

In regard to cancer patients with VTE, the ESMO guideline considers that the efficacy and safety of the DOACs that inhibit the Xa factor are an alternative to LMWH, but in obese patients, this guideline continues to recommend LMWH above DOAC. 50

##### Recommendations

- In patients with obesity, DOAC can be used when the BMI is <40 kg/m ^2^ or in individuals with a weight <120 kg (Grade III, C recommendation).
- If treatment with DOAC is unavoidable, standard doses of rivaroxaban or apixaban can be used in patients with a BMI >40 kg/m ^2^ or in individuals with a weight >120 kg (Grade V, C recommendation).
- It is suggested not to use DOAC for the treatment or prevention of VTE in the acute phase following bariatric surgery, and instead, the use of parenteral anticoagulation in the post-surgical phase is recommended (Grade IV, B recommendation).
- In patients with cancer-associated thrombosis and obesity, the use of LMWH is recommended above the use of DOAC (Grade III, B recommendation).

### Therapeutic Management of Older Patients with Pluripathology and Polymedication

It is estimated that two-thirds of the cases of VTE are found in patients over 70 years of age. 54 In addition to the physiopathological changes associated with aging, the clinical conditions related to age that may influence the risk of VTE must be added as well as the safety and adherence to anticoagulant treatment: RI, body composition, falls, cognitive decline, polypharmacy, cancer, etc. 55 56

Aging is highly heterogeneous; the same chronological age may conceal different frailty profiles. Comprehensive Geriatric Assessment (CGA) is a multidimensional clinical evaluation that allows defining the frailty profile to have prognostic and predictive value of complications. The CGA includes geriatric interventions for conditions susceptible to improvement or resolution: comorbidity, polypharmacy (interactions), nutritional status (body composition, malnutrition), cognitive and emotional status, and social support. 57

There are no specific guidelines of therapeutic management in older patients, but rather the recommendations derived from clinical trials in which this population is underrepresented are followed. There is little evidence on the safety of treatment with LMWH in geriatric patients, although a review that analyzed the efficacy and safety of treatment with doses adjusted to weight showed no increase in the risk of bleeding in relation to age. 58 Since the elimination of LMWH is mainly renal, maintained treatment with LMWH could expose older patients with diminished renal function to a potential risk of drug accumulation and a consequent higher risk of bleeding. Two studies on different LMWHs reported pharmacokinetic differences. While one study demonstrated the accumulation of nadroparin at 10 days of treatment in relation to creatinine levels and anti-Xa activity, the other study including patients with a median age of 85 years treated with weight-adjusted tinzaparin showed no correlation between anti-Xa activity and age, weight, or renal function. 59 60

The median age of the patients included in the four main studies comparing DOAC versus standard therapy with VKA was 55 to 60 years, and none of the studies considered age as a factor for adjusting the treatment dose. 61

A meta-analysis of the subgroup of patients ≥75 years of age demonstrated that DOACs are more effective and safe than VKA, with no increase in the risk of bleeding. 61 In the EINSTEIN-DVT/PE studies, the risk of major bleeding was much lower in patients receiving rivaroxaban compared with standard therapy. 62 In the HOKUSAI study, edoxaban demonstrated to be more effective than warfarin in the subgroup of frail patients, with no differences in the risk of bleeding. 63

A retrospective study of the RIETE (Computerized Registry of Patients with Venous Thromboembolism) registry concluded that in patients >80 years of age, the incidence of thrombotic events surpasses the risk of fatal hemorrhage during anticoagulant treatment. 64 However, prolonging the treatment beyond the third month was associated with a greater risk of major bleeding than recurrence of PE. 65

The 2016 ACCP guidelines recommend performing a risk/benefit assessment of anticoagulant treatment in patients over 75 years of age and in those more than 65 years old with a risk of falls and low risk VTE (isolated distal VTE or subsegmentary pulmonary thromboembolism secondary to a transitory risk factor) to decide whether to administer anticoagulation or maintain a conservative attitude. 52

In patients with idiopathic VTE and a high risk of bleeding, 3 months of anticoagulation is recommended, while in patients with cancer-associated thrombosis or idiopathic VTE with moderate or low risk of bleeding, it is suggested to maintain the treatment indefinitely. 52

#### Recommendations

How should the geriatric assessment be incorporated in the individualization of anticoagulant treatment?

- The indication of anticoagulant treatment in older patients should include comprehensive geriatric assessment (CGA) to define the frailty profile and monitor the risk of recurrence and bleeding, and treatment adherence. Among the variables that make up the CGA, comorbidity, polypharmacy, functional status, cognitive status, and social support are of special relevance. CGA includes the planning of geriatric interventions specifically aimed at potentially improvable or reversible risk factors (Grade IV, C recommendation).

How can thrombotic and hemorrhagic complications be minimized?

- It is recommended to reduce polypharmacy with the aim of decreasing potential interactions with oral anticoagulants. Avoid pharmacokinetic interactions between DOAC and CYP3A4 or P-glycoprotein inhibitors/inducers. In cases with polypharmacy and a high risk of drug interactions, the use of LMWH is preferred, with a periodic reassessment of the risk–benefit of the treatment (Grade IV, C recommendation).
- Renal function must be monitored in older patients to adequately adjust the dosage of DOAC or LMWH. DOACs are contraindicated if creatinine clearance is <15 mL/min (Grade IV, B recommendation).
- In patients with little mobility and/or risk of falls, measures can be taken to improve their physical status and adapt the environment to attempt to avoid falls (Grade V, C recommendation).
- To guarantee treatment adherence, the autonomy of treatment management of the patient and/or the identification of a responsible caregiver must be assessed. It is important for the patient and/or main caregiver to receive clear information regarding the disease and the treatment (Grade V, C recommendation).

Is age a limitation on the time anticoagulant treatment should be maintained?

Age, itself, is not a factor to contraindicate anticoagulant treatment.

1. In older patients with a high risk of bleeding or low risk of thrombotic recurrence (VTE secondary to a transitory risk factor or distal DVT) 3 months of treatment should be considered (Grade III, C recommendation).
2. If the risk of bleeding is not high, in older patients with idiopathic or cancer-associated VTE, long-term treatment should be considered with periodic risk–benefit reassessments (Grade I, B recommendation).
3. In long-term treatment (>6 months) with apixaban and rivaroxaban, dose reduction is suggested (Grade II, C recommendation).

### Primary Thromboprophylaxis in Ambulatory Cancer Patients with Systemic Antineoplastic Treatment

Cancer patients present an increased risk of VTE, estimating that approximately between 4 and 20% of patients with cancer present VTE at some time. 66 In the last two decades the accumulated incidence of cancer-associated VTE has tripled. 67 Approximately 80% of all the events occur in non-hospitalized patients. 68

The incidence of thrombosis in these patients varies widely, and thus, it is important to identify the patients with greater risk of developing VTE in whom thromboprophylaxis may be beneficial. Recently, an incidence of VTE greater than 30% has been described in patients with pancreatic cancer and in specific molecular subtypes of non-small cell lung cancer with ROS-1 and ALK rearrangement. 69 70 71 72

In the last decades, different approaches have emerged for stratifying the risk of VTE in cancer patients with risk assessment models (RAM). Among these, the Khorana score was the first to be developed. It has been validated in multiple settings and its application is recommended by most international guidelines. However, its sensitivity is low, and its reproducibility is limited in specific types of cancer. Although other models have been developed in the last decade, such as the Vienna CATS, PROTECHT, CONKO, ONKOTEV, and COMPASS-CT, most have obtained poor results in validations of external cohorts. 73 74

Risk models with more novel approaches have recently been developed and include the prediction of personalized risk based on continuous biomarker levels, 75 the search for innovative biomarkers such as genetic polymorphisms, 76 or the use of machine learning techniques and natural language processing 73 ( Table 7 ).

**Table 7:** Risk assessment models (RAM) for cancer-associated thrombosis

RAM	Variables	Proposed cut-off	External validation
**Khorana score**	• Very-high risk tumor types a (2) • High-risk tumor types b (1) • Hemoglobin <10 g/dL (1) • Platelet >350/10 ^9^ /L (1) • Leucocyte >11/10 ^9^ /L (1) • BMI ≥35 kg/m ^2^ (1)	≥3 vs. 1–2	Yes
**CATS score**	• Very-high risk tumor types (2) • High risk tumor types (1) • Haemoglobin < 10 g/L (1) • Platelet > 350/10 ^9^ /L (1) • Leucocyte > 11/10 ^9^ /L (1) • BMI ≥ 35 Kg/m ^2^ (1) • D-Dimer ≥ 1.44 µg/L (1) • P-selectin ≥ 53.1 ng/L (1)	≥ 3 vs 0–2	Yes
**PROTECHT**	• Very-high risk tumor types (2) • High-risk tumor types (1) • Hemoglobin <10 g/L (1) • Platelet >350/10 ^9^ /L (1) • Leucocyte >11/10 ^9^ /L (1) • BMI ≥35 kg/m ^2^ (1) • Gemcitabine (1) • Platinum (1)	≥3 vs. 0–2	Yes
**CONKO score**	• Very-high risk tumor types (2) • High-risk tumor types (1) • Hemoglobin <10 g/L (1) • Platelet >350/10 ^9^ /L (1) • Leucocyte >11/10 ^9^ /L (1) • ECOG-PS ≥2 (1)	≥3	Yes
**COMPASS-CT score**	• Anti-hormonal therapy/anthracycline (6) • Cardiovascular risk factors c (5) • Recent hospitalization/acute medical illness (5) • ≤6 months since cancer diagnosis (4) • Central venous catheter (3) • Advanced/Metastatic disease (2) • Prior VTE (1) • Platelet >350/10 ^9^ /L (2)	≥7	Yes
**ONKOTEV score**	• Khorana >2 (1) • Previous VTE (1) • Metastasis (1) • Vascular/lymphatic macroscopic compression (1)	>2	Yes
**TIC-ONCO score**	• BMI >25 • Family VTE history • Primary tumor site • Tumor stage • GRS	Different cut-offs based on sensitivity	Yes
**CT nomogram**	• D-dimer (continuous) • Tumor-site risk	Personalized risk prediction	Yes
**LI score**	• Colorectal cancer (1) • High-risk tumor types (2) • Very-high risk tumor types (3) • Pretherapy BMI ≥35 (1) • Pretherapy leucocyte >11 (1) • Pretherapy hemoglobin <10 g/L (1) • Pretherapy platelet ≥350/10 ^9^ /L (1) • Cancer staging III–IV (1) • Targeted or endocrine monotherapy (−1) • History of VTE (1) • History immobility in past 12 months (1) • Recent hospitalization >3 days past 3 months (1) • API (Asian/Pacific Islander) race (−1)	>2	No

Abbreviations: BMI, body mass index; ECOG-PS, Eastern Cooperative Oncology Group - Performance Status; GRS, genetic risk score; VTE, venous thromboembolism.

Notes: ^a^ Very-high risk tumors; pancreatic, gastric, esophageal, gallbladder.

b High-risk tumors: lung, lymphoma, gynecologic, bladder, testicular and brain.

c Cardiovascular risk factors: two or more of the following: history of peripheral artery disease, ischemic stroke, coronary artery disease, hypertension, hyperlipidemia, diabetes, obesity.

At present, international guidelines do not recommend routine thromboprophylaxis for all cancer outpatients given the great heterogeneity, but do recommend its use in patients with very high risk as well as in specific patient profiles of medium and high risk, assessing the risk of bleeding ( Table 8 ). 46 50 77 78 79 80

**Table 8:** Summary of guideline recommendations addressing primary prophylaxis in ambulatory cancer patients

Guideline	Recommendations
**ESMO 2023**	• VTE risk assessment should be based on validated RAMs such as the KRS, COMPASS-CT score, or CT nomogram (III, C). An estimated risk of VTE >8–10% at 6 months is suggested as threshold for discussing primary thromboprophylaxis (II, C). • For ambulatory pancreatic cancer patients on first-line systemic anticancer treatment, LMWH given at a higher dose for a maximum of 3 months may be considered (II, C). • In ambulatory cancer patients starting systemic anticancer treatment who have a high thrombosis risk, apixaban, rivaroxaban, or LMWH may be considered for primary thromboprophylaxis for a maximum of 6 months (I, B). • Where concerns of DOAC safety exist and the patient is perceived as having clinically important risk for VTE, LMWH at conventional primary thromboprophylaxis dosing may be administered (II, C).
**ASCO 2023**	• Routine pharmacologic thromboprophylaxis should not be offered to all outpatients with cancer (strong recommendation). • High-risk outpatients with cancer (Khorana score of 2 or higher before starting a new systemic chemotherapy regimen) may be offered thromboprophylaxis with apixaban, rivaroxaban, or LMWH provided there are no significant risk factors for bleeding and no drug interactions (moderate recommendation). • Patients with multiple myeloma receiving thalidomide- or lenalidomide-based regimens with chemotherapy and/or dexamethasone should be offered pharmacologic thromboprophylaxis with either aspirin or LMWH for lower risk patients and LMWH for higher risk patients (strong recommendation).
**NCCN 2023**	• VTE risk evaluation based on Khorana score of patients with cancer receiving/starting systemic therapy for their cancer. • Consider anticoagulant prophylaxis (LMWH or DOAC) for up to 6 months or longer if risk persists in patients with intermediate or high risk for VTE (Khorana score ≥2). Patients with gastric or gastroesophageal tumors are at increased risk for hemorrhage with DOAC.
**ITAC/ISTH 2022**	• Primary pharmacological prophylaxis of VTE with LMWH (grade 1A) or with DOAC (rivaroxaban or apixaban; grade 1B) is indicated in ambulatory patients with locally advanced or metastatic pancreatic cancer treated with systemic anticancer therapy and who have a low risk of bleeding. • Primary prophylaxis with DOAC (rivaroxaban or apixaban) is recommended in ambulatory patients who are receiving systemic anticancer therapy and are at intermediate to high risk of VTE, identified by a validated risk assessment model (i.e., a Khorana score ≥2), and not actively bleeding or not at a high risk for bleeding (grade 1B). • In patients with myeloma treated with immunomodulatory drugs combined with steroids or other systemic anticancer therapies, VTE primary pharmacological prophylaxis is recommended (grade 1A); in this setting, oral anticoagulants (vitamin K antagonists at low or therapeutic doses and apixaban at prophylactic doses), LMWH at prophylactic doses, or low-dose aspirin (100 mg daily) can be used and have shown similar effects with regard to preventing VTE (grade 2B). Values and preferences: subcutaneous injections.
**ASH 2021**	• Classification of risk based on validated risk assessment tool (i.e., Khorana score) complemented by clinical judgment and experience. • Low risk: no thromboprophylaxis recommended (over LMWH)/suggested (over DOAC). • Intermediate risk: apixaban/rivaroxaban or no thromboprophylaxis suggested. • High risk: apixaban/rivaroxaban or LMWH suggested. • For multiple myeloma patients receiving lenalidomide-, thalidomide-, or pomalidomide-based regimens, the ASH guideline panel suggests using low-dose ASA, fixed low-dose VKA, or LMWH.
**SEOM 2019**	• It is recommended to use a validated RAM to assess VTE risk (level of evidence: grade 2C). • Routine thromboprophylaxis is not recommended in ambulatory patients with cancer (level of evidence: grade 1B). • Pharmacological thromboprophylaxis with LMWH or DOACs may be considered in high-risk ambulatory cancer patients, such as advanced pancreatic cancer, NSCLC with ROS-1 or ALK rearrangement, patients with a Khorana score ≥2 or considered high risk based on a validated RAM, starting of receiving systemic therapy, and no contraindications to anticoagulation and low risk of bleeding. Pharmacological thromboprophylaxis is suggested at least 12 weeks after the initiation a new systemic therapy. Perform DDI assessment with DOAC. Discuss with patient potential risk and benefits (level of evidence: grade 1B).

Abbreviations: ASA, acetylsalicylic acid; ASCO, American Society of Clinical Oncology; ASH, American Society of Hematology; DDI, drug–drug interaction; DOAC, direct oral anticoagulant; ESMO, European Society for Medical Oncology; ISTH, International Society on Thrombosis and Haemostasis; ITAC, International Initiative on Thrombosis and Cancer; KRS, Khorana score; LMWH, low molecular weight heparin; NCCN, National Comprehensive Cancer Network; NSCLC, non-small cell lung cancer; RAM, risk assessment model; SEOM, Spanish Society of Medical Oncology; VTE, venous thromboembolism.

Different randomized clinical trials and meta-analyses comparing anticoagulant prophylaxis with no intervention or with placebo have evaluated the role of primary thromboprophylaxis with LMWH in ambulatory patients receiving cancer treatment. In general, a significant 53% reduction was observed in the rate of incidence of VTE in patients with intermediate and high risk (Khorana score ≥2) without a significant increase in the risk of major bleeding. 81 This reduction was greater in patients with pancreatic cancer (82–74%) 82 and in patients with lung cancer (58%). 83

Two randomized clinical trials have specifically studied the safety and efficacy of direct Xa factor inhibitors, rivaroxaban and apixaban, in comparison with placebo during a 6-month period in patients with intermediate and high risk (Khorana score ≥2). On analyzing the two studies together in a meta-analysis, the reduction in the RR of VTE of the DOAC versus placebo seemed to be slightly lower (43%) than in the studies of LMWH, but they were associated with a non-significant increase in the risk of major bleeding (RR, 1.96). 81

In the same systematic review and meta-analysis, it was concluded that in ambulatory cancer patients with intermediate and high risk, thromboprophylaxis with DOAC or LMWH significantly reduces the risk of VTE (number needed to treat [NNT], 25) without a significantly greater risk of major bleeding (number needed to harm, 1,000). 81

In a systematic review evaluating the efficacy and safety of primary thromboprophylaxis in ambulatory pancreas cancer patients receiving chemotherapy, it was found that primary thromboprophylaxis with anticoagulants was associated with a reduction in the RR of 69% in the rates of VTE, resulting in an NNT of 11.9 for preventing an event of VTE, without increasing the risk of major bleeding. 84

The duration of pharmacological thromboprophylaxis in ambulatory cancer patients cannot be determined with certainty. The first 3 months after the diagnosis and the initiation of cancer treatment constitute the conventional period of greatest risk during which >50% of VTE events occur, and all the studies available have covered at least this period. 50 On the other hand, the 2023 guideline of the National Comprehensive Cancer Network (NCCN) states that thromboprophylaxis can be prolonged to 6 months or more if the risk is maintained. 80

#### Recommendations

How should the risk of thrombosis be assessed in ambulatory cancer patients?

- The evaluation of risk of VTE should be based on the use of any of the validated RAM at the time the patient initiates systemic antineoplastic treatment (Grade III, C recommendation).
- As a threshold for considering primary thromboprophylaxis, an estimated risk of VTE of >8 to 10% at 6 months is suggested (Grade II, C recommendation).

In what patient profile should pharmacological thromboprophylaxis be performed?

- Primary pharmacological prophylaxis of VTE should be performed with LMWH (Grade I, A recommendation) or with DOAC (rivaroxaban or apixaban; Grade II, B recommendation) in ambulatory pancreas cancer patients with locally advanced or metastasis treated with systemic antineoplastic therapy and who have a low risk of bleeding.
- Consider performing pharmacological thromboprophylaxis with LMWH or DOAC in other patients with high risk, such as those with non-small cell lung cancer with ROS-1 or ALK rearrangement, and patients classified as high risk according to a validated RAM, who are receiving systemic therapy, without contraindications for anticoagulation and with low risk of bleeding (Grade II, B recommendation).
- Consider performing at least 12 weeks of thromboprophylaxis after the initiation of a new systemic therapy (Grade II, B recommendation).
- Special caution is suggested with the use of DOAC in patients with a high risk of bleeding (gastrointestinal/genitourinary tumors, and comorbidities which increase the risk) and in patients with potential pharmacological interactions (Grade I, B recommendation).
